# Management of Breech Presentations1Read at the meeting of the American Association of Obstetricians and Gynecologists, Washington, D. C., September 16, 17 and 18, 1902.

**Published:** 1903-01

**Authors:** William D. Porter

**Affiliations:** Cincinnati, O.


### Management of Breech Presentations.[1]

By WILLIAM D. PORTER, M. D., Cincinnati, O.

(*American Journal of Obstetrics, December, 1902.*)

BREECH presentation is encountered once in about fifty cases of labor. A much higher ratio represents its pro rata agency in inflicting damage on the mother, in causing injuries to the child, and particularly in the production of fetal mortality. The mortality is variously estimated at from 10 to 35 per cent. The percentage of children that die in the twenty-four hours succeeding birth is also high. It is difficult to determine what proportion of the deaths is due to the presentation *per se.* Many of the labors are premature. A change from breech to head presentation occurs late in some pregnancies. In case of premature labor with breech presentation, dystocia incident to the presentation may cause death of the child. It may be due wholly to the premature labor, which anticipated a possible change to a vertex presentation. But it must be admitted that the presentation may cause the premature labor. The breech seldom enters the pelvis in advance of labor, which may be precipitated from lack of space. The principle is the same as that which explains the frequency of premature labors in cases of contracted pelves.

Primiparity is an important factor of fetal mortality. The abdominal wall admits of less distension, making premature

1. Read at the meeting of the American Association of Obstetricians and Gynecologists, Washington, D. C., September 16, 17 and 18, 1902.

labor more probable. The soft parts dilate less readily. The vagina is less capacious. These conditions increase the difficulty both of labor and of manipulations, should the latter be needed.

Breech cases always require careful supervision and frequently there is urgent need of assistance. On the contrary, it is equally true that defective judgment as to whether interference is needed, and inferior skill in rendering aid, contribute in an unfortunate degree to the fetal mortality.

Breech cases are usually tedious. The position of the child is generally oblique. Its pelvis, held by the comparatively rigid portion of the spinal column, does not readily accommodate itself to the resultant of forces, as does the head under like conditions. Consequently the breech is likely to impinge against the brim near the anterior end of an oblique diameter. Much time and energy may be consumed in overcoming this obstacle.

The child's thighs are usually flexed. That the breech may engage, this flexion must be carried to an extreme degree. This is opposed by the resiliency of the tissues, reinforced doubtless by resistance on the part of the voluntary muscles. At a definite point in the labor the uterine efforts are expended in crowding the breech into the brim against this resistance. Promptly on the cessation of the pain the extension of the thighs lifts the breech out of the pelvis. The next pain is expended in doing the same work, which is as promptly undone. This seesaw will continue until finally the breech is driven so deep into the pelvis that the thighs are parallel with the body, and efforts at extension only serve to fix the breech more firmly.

The progress of the breech through the pelvis is necessarily slow. However, by the time the outlet is reached the distension of the vagina and vulva is so great that the reflexes stimulate the expulsive powers to their greatest effort. The reflexes are greater than when the head presents, owing to the greater mass within the vagina. Expulsive efforts of such power and rapidity are produced that the child is likely to be rapidly expelled, reducing its time of greatest peril to a minimum.

Serious delay in a breech case is likely to be at one of three points: (1) with the breech at the brim; (2) with the breech in the pelvis; (3) with the delivery of the head.

Delay of the breech at the brim may become a serious matter, particularly if the membranes are ruptured. The water readily escapes, and retraction of the uterine walls may occur to a degree sufficient to embarrass the uteroplacental circulation. This is always a cause for apprehension, and particularly so in breech cases. For there comes a time when this circulation is suspended, owing to the compression of the cord by the chest and head as they pass through the pelvis. The ability to meet this ordeal depends largely on how well the blood is oxygenated before the ordeal begins. The principle is well exemplified in divers who give exhibitions which involve remaining under water for a comparatively long time. It is noticeable that, before undertaking such a feat, the blood is well oxygenated by a number of deep rapid respirations.

With the breech delayed at the superior strait, the usual teaching is to bring down one or both legs and make traction. This plan is excellent if the patient be a multipara and the cervix well dilated or easily dilatable. In case of a primipara, or of a multipara with the cervix imperfectly dilated and with evidence that the completion of the dilatation will require considerable force, it is a questionable procedure. With one or both legs in advance a wedge is formed whose sides taper so gradually as to form a powerful dilator. But its maximum diameter is insufficient to prepare the soft parts for the prompt passage of the head. If the resistance of the cervix is considerable, it will promptly contract when the shoulders have passed, and may cause a fatal delay of the head or a serious laceration of the cervix. Should the perineum form the chief resistance, the delivery of the head within a safe time limit will probably result in an extensive tear of the perineum and possibly of the vagina.

If greater dilatation can be safely secured before the head reaches the pelvis, there will be a manifest gain of possibly vital importance. Such a gain is inevitable if the thighs be allowed to remain flexed and the legs extended along the body of the child. If the breech is carried through the cervix and over the perineum, with the legs in this position, the dilatation is generally sufficient to permit the ready extraction of the head. Considerably more time is consumed by this plan than is needed if the legs are brought down. If interference has not been too long delayed this extra time is well spent. Minutes spent in extricating the head count for more than hours expended in bringing down the doubled breech. The cervix dilated in the slower manner, by the larger mass, is not so likely to contract about the child's neck. It would doubtless be better to do nothing in preference to bringing down the legs, though such an election would often be but the choosing of the lesser evil.

William Hunter, whose contemporary fame equaled that of his brother, the great John Hunter, was an able obstetrician and a noted teacher. At one time he advised bringing down the legs in the condition under discussion. He reasoned that by lessening the mass the dilatation would be easier and more gradual, and that the suffering of the woman would thereby be greatly lessened. In his later lectures he admitted the danger of his plan and advised strongly against it. In nearly every case in which he had brought down the legs he lost the child.

The best method of bringing down the doubled breech is to make traction over the anterior groin with a finger. In case of a multipara this is easily accomplished, particularly if only moderate traction is needed. If traction in this manner is not feasible or is inadequate, a broad tape or a handkerchief carried between the anterior thigh and the body of the child furnishes an efficient means of traction.

The objection is made that it is sometimes difficult to place the fillet in position. It is no more, or but little more, difficult than to bring down a leg. In placing the fillet the Trush forceps will be found a valuable instrument. The objection that injury has been done with the fillet is applicable to all obstetrical instruments and manipulations. If traction be made in the proper direction the force is expended largely on the pelvis rather than on the femur. Moderate traction in conjunction with the pains will, as a rule, insure gradual descent.

Delay may occur with the breech in the pelvis. Almost invariably the thighs are flexed. The mass is about as great as the pelvis can accommodate. It does not readily bend and adjust itself to the pelvic curves. This is partly due to the resistance of the inflexible thighs. Uterine inertia from exhaustion is also a possible cause of delay.

Robert Barnes taught[1] that delay, with the breech low, often gave rise to serious difficulty. He advised against direct traction on the breech in all cases, claiming that it is frequently unsuccessful, and if so, does harm. His treatment was always to bring down a leg. This procedure he terms breaking up or decomposing the obstructing wedge. With the breech low in the pelvis it is difficult to bring down a leg. And notwithstanding the high authority, it would seem to be unnecessary.

The use of forceps on the breech is advocated by men of recognised ability. If only slight traction be needed they are

1. Obstetric Operations.

doubtless safe and satisfactory. They are so poorly adapted to the shape of the pelvis that it is difficult to regard them as appropriate in cases demanding much assistance. Traction on the groin with the finger, or, if necessary, with the fillet, seldom fails to overcome the delay.

As the body of the child is delivered it should be enveloped in a warm towel. This insures a secure grasp, conserves the child's heat, and lessens the danger of premature efforts at respiration.

As the body is extracted it is well to remember that the shoulders should enter an oblique diameter of the pelvis. To keep the spinal column directly under the pubic arch, as is pictured in many of the textbooks, is to force the shoulders to enter the transverse diameter, with the not remote danger of fracturing a clavicle.

Among the various procedures for delivering the after-coming head, that known as the Deventer method has the sanction of good authority. With the woman in the lithotomy position, as soon as the shoulders appear, traction vertically downward is made, one hand grasping the feet and the other making traction over the shoulders. The arms are undisturbed and are usually found placed along the sides of the head. The claim that by this method the head is delivered in full extension is hardly credible. The normal position of the head is that of flexion, due to the pronounced curve of the spinal column of the child *in utero*. In this position of flexion the head will in all probability enter the pelvis, unless extended by awkward efforts at suprapubic pressure. The explanation of Reynolds[1] that the chin is arrested on the pelvic floor, causing the head to become extended, is not tenable. It may be stated as an inflexible law that, with the head in the pelvis, a change from flexion to extension or from extension to flexion is a physical impossibility.

The so-called Prague method, but which Parvin attributes to Puzos, involves traction by the feet and shoulders, first downward and backward till the head enters the pelvis; then upward and forward, bringing the back toward the mother's abdomen as the head is delivered.

"The combined method" seeks to diminish traction on the neck and to insure flexion by pressing two fingers against the superior maxilla. This method is variously attributed to Smellie, to Veit, and to Mauriceau. Others claim to effect the

1. American Textbook of Obstetrics.

same results by traction on the inferior maxilla with one or two fingers in the mouth.

Pressure over the fundus is a most valuable adjunct, whatever method be selected.

A matter of great moment is the amount of traction which can safely be exerted through the neck. This is estimated by Matthews Duncan, from experimental data, to be about 105 pounds. Goodell, from clinical experience, placed the estimate still higher. I have seen fatal injuries to the soft structures of the neck with much less traction than the estimates above given. Where such tremendous force is necessary the forceps is certainly safer and its application is a matter of seconds. For even with an obstruction at the brim, we must remember that most of the head enters the pelvis before resistance is encountered. In this condition the application of forceps is easy.

The method of delivering the head which I have found most satisfactory (though it has no original features) is as follows: After delivery of the breech, bring down the legs and grasp them, enveloped in a towel, with the left hand, making traction. Make pressure over the fundus with the right hand. The importance of this pressure cannot be overestimated. To get the best results from such pressure the woman should be anesthetised. When the shoulders appear the arms should be swept down over the face. As the head enters the pelvis it should be crowded down with the hand, keeping the pelvic curve in mind. If additional force is needed an assistant may make pressure over the fundus. Ordinarily the pressure and traction can be better timed and regulated by one person than by two. Moreover, the proper axis in which to make traction can be more accurately determined with this bimanual method. And, with all respect for the opinions of those who attribute failure to the lack of sufficient flexion, it appears more rational to explain it by traction in a wrong axis.

The possibility of a large head or of a pelvic contraction should be anticipated by having the forceps always in readiness. If there is reason to believe that great traction will be necessary, the patient should be placed on a table of good height. The advantage of occupying a seat lower than the hips of the patient is equaled only by the satisfaction of having a firm, unyielding support for the hips. With the hips on an ordinary mattress it is necessary to vary the direction of traction with every change in the amount of traction.

To consume time in attempting to supply air to the child before delivery of the head is to forget what should be an obstetrical motto: "If in a given case it is possible to deliver the after-coming head, it can and must be done with promptness."

